# The impact of transcranial magnetic stimulation on serum thyroid-stimulating hormone levels in depressive patients: a systematic review and meta-analysis of randomized controlled trials

**DOI:** 10.3389/fpsyt.2026.1716377

**Published:** 2026-01-22

**Authors:** Yutai Ma, Chunyan Wei, Qiao Pan, Yali Wang

**Affiliations:** 1 School of Health Management, Gansu University of Chinese Medicine, Lanzhou, China; 2 Department of Psychology, The Third People’s Hospital of Lanzhou, Lanzhou, China

**Keywords:** depression, meta-analysis, randomized controlled trials, thyroid-stimulating hormone, transcranial magnetic stimulation

## Abstract

**Introduction:**

The findings of current research on the impact of transcranial magnetic stimulation (TMS) on thyroid function are inconsistent. This study investigated the effects of TMS on the function of the hypothalamic-pituitary-thyroid axis (HPT axis) in patients with depression through meta-analysis, with a focus on the thyroid-stimulating hormone (TSH).

**Methods:**

From inception to November 27, 2025, we conducted a systematic search of randomized controlled trials investigating TMS therapy for depression across PubMed, Embase, Web of Science, the Cochrane Library, and Chinese biomedical databases. Two independent reviewers screened the retrieved abstracts and full-text articles using our predefined search strategy, in duplicate, to assess eligibility criteria, extract data, and evaluate the risk of bias. Due to the high heterogeneity, a random-effects model was selected. The review protocol was prospectively registered in PROSPERO (CRD420251006441).

**Results:**

Meta-analyses of the 5 randomized controlled trials included demonstrated that TMS treatment had no statistically significant effect on TSH levels in depressed patients (Z = 0.06, P = 0.952). Sensitivity analyses and publication bias tests(Egger’s regression test, t = 1.01, P < 0.386) supported the robustness of the results. TSH levels in postpartum depressed patients were also not statistically significantly modulated by TMS (Z = 1.71, P = 0.087), but the results were not robust.

**Discussion:**

This meta-analysis indicates that TMS exerts no statistically significant influence on TSH concentrations in individuals with depression, implying a negligible effect on the HPT axis. However, it is important to emphasize that this constitutes only initial preliminary evidence. Although the findings are consistent across the depressive cohort, the data about postpartum depression remain inconclusive, highlighting the necessity for additional investigation. These results enhance our understanding of TMS’s neuroendocrine mechanisms within the context of depressive disorder.

**Systematic Review Registration:**

https://www.crd.york.ac.uk/PROSPERO/view/CRD420251006441, identifier CRD420251006441.

## 1 Introduction

The primary features of depression include persistent and severe mood disturbances. Affected individuals often exhibit dysregulation of the hypothalamic-pituitary-thyroid (HPT) axis (1, 2). The impact of physical therapies widely used in depression treatment on the HPT axis remains unclear. Transcranial magnetic stimulation (TMS) is a non-invasive neurostimulation technique that uses pulsed magnetic fields to induce electrical currents in the cerebral cortex. This process modulates neuronal activity, thereby regulating cerebral metabolism and neurofunction (3). Since its approval by the U.S. Food and Drug Administration (FDA) in 2008 for the treatment of depression, TMS has become a significant physical intervention for treatment-resistant depression (4, 5). The 2016 expert consensus from the International TMS Society further substantiates its clinical utility (6–8). However, current research on the effects of TMS on the HPT axis exhibits considerable heterogeneity.

The heterogeneity of existing research findings is substantial, and there is currently no systematic review or meta-analysis that consolidates the quantitative evidence. Kito (9) found that after TMS treatment in the right dorsolateral prefrontal cortex (DLPFC), responders had significantly higher thyroid-stimulating hormone (TSH) levels but no significant changes in free triiodothyronine (FT3) and free tetraiodothyronine (FT4). And Meille (10) found that after TMS treatment in the right DLPFC, no significant TSH, FT3, or FT4 changes were observed in either responders or non-responders. Meige Sun (11) reported that TMS treatment in the left temporoparietal cortex increased TSH, FT3, and FT4 levels; and Zizhen Huang (12) found that TMS treatment in the left DLPFC decreased TSH but did not affect FT3/FT4 levels.

We selected TSH as the focal point and primary outcome of this meta-analysis for the following reasons. First, from a pathophysiological perspective, TSH is the most sensitive and central regulator of the HPT axis. It serves as the primary screening biomarker for thyroid dysfunction in both clinical and research settings because its levels change dynamically in response to even subtle perturbations in thyroid homeostasis, often before alterations in circulating FT3 or FT4 become detectable (13). Second, from a methodological and pragmatic standpoint, among the limited RCTs that measured any HPT-axis hormones, TSH was the most commonly reported thyroid function marker. While assessing a broader panel of hormones (e.g., FT3, FT4, thyrotropin-releasing hormone (TRH)) would provide a more comprehensive view, the available data for these parameters were insufficient for a reliable meta-analysis at present. Therefore, focusing on TSH provides a critical and feasible first step to consolidate the existing evidence regarding TMS’s potential influence on the neuroendocrine system.

We conducted this systematic review and meta-analysis to integrate the existing evidence on the effects of TMS on TSH in depressed patients. Our primary aims were to clarify the characteristics of TMS-induced modulation of the HPT axis and to establish a comparative framework with the mechanism of action of antidepressants. This comparison will provide a new perspective for elucidating the neuroendocrine mechanisms of antidepressant treatments.

## 2 Methods

### 2.1 Inclusion and exclusion criteria

Inclusion Criteria (1): Patients diagnosed with depression according to standardized diagnostic guidelines (ICD-10, DSM-III/IV/V, CCMD-3) or expert consensus (2); Intervention comparison: Experimental group receiving TMS combined with conventional treatment; Control group receiving conventional treatment alone (3); Studies reporting pre- and post-treatment serum TSH levels or change scores (4); Randomized controlled trial (RCT) design. Exclusion Criteria (1): Dissertations or thesis publications (2); Studies with incomplete data or duplicate publications (3); Articles lacking full-text access (4); Patients with comorbid thyroid disorders or on thyroid hormone therapy.

### 2.2 Study selection

A systematic review and meta-analysis were conducted following the Preferred Reporting Items for Systematic Reviews and Meta-Analyses (PRISMA) guidelines (14–16). Comprehensive electronic searches were performed across PubMed, Embase, Web of Science, Cochrane Library, China Biology Medicine disc (CBM), China National Knowledge Infrastructure (CNKI), China Science and Technology Journal Database (CSTJ), and Wanfang Data from inception through November 27, 2025. The search strategy combined subject headings and free-text terms related to depression, thyroid-stimulating hormone, and transcranial magnetic stimulation (see Supplementary_Material for search strategy). The review protocol was registered prospectively in PROSPERO (CRD420251006441) (17, 18). Retrieved records were de-duplicated using EndNote, and two independent reviewers (Yutai Ma and Qiao Pan) screened titles, abstracts, and full texts. Discrepancies were addressed through consultation with Chunyan Wei, and the reference lists of included studies were meticulously reviewed to identify supplementary relevant research.

### 2.3 Data extraction

Two independent reviewers (Yutai Ma and Qiao Pan) systematically extracted relevant data into a structured Excel database, encompassing study characteristics (author, publication year, source, study design), participant demographics (sample size, proportion of female participants, mean age), country of origin, diagnostic criteria, merger situations, washout period, and stimulation site. Discrepancies in data extraction were resolved through discussion with Chunyan Wei. For ambiguous or missing information, correspondence was established with the original study authors for clarification.

### 2.4 Statistical analyses

The primary outcomes involved continuous variables, analyzed using standardized mean difference (SMD) to accommodate heterogeneity in TSH assessment methodologies across studies. In cases where studies employed uniform TSH measurement protocols, weighted mean difference (WMD; Cohen’s d) was utilized (19, 20). Effect estimates are presented with 95% confidence intervals (CIs). Model selection between random-effects (RE) and fixed-effects (FE) was based on the degree of heterogeneity observed (21, 22). The random-effects model was chosen *a priori* for all meta-analyses due to the anticipated clinical and methodological heterogeneity across studies. This choice was subsequently confirmed by the observed high statistical heterogeneity (I² = 97%, p < 0.001; I²=92.7%, p<0.001). Meta-analyses comparing TSH level changes between groups were performed using StataSE. Publication bias was assessed via funnel plot visualization (23, 24) and Egger’s regression test. Meta-regression analyses were conducted on sample size, number of females, age, treatment duration, TMS stimulation regions and frequency, and concomitant antidepressant to explore sources of heterogeneity. Sensitivity analyses employing the leave-one-out approach were performed to evaluate the robustness of pooled estimates.

### 2.5 Risk of bias assessment

Two independent reviewers (Yutai Ma and Qiao Pan) evaluated the risk of bias for each included study utilizing the Cochrane Risk of Bias 2 (ROB2) tool (25, 26). The assessment encompassed five domains: randomization process, deviations from intended interventions, missing outcome data, outcome measurement, and selective reporting. Judgments were recorded in Excel as “Low risk,” “Some concerns,” or “High risk.” Any discrepancies were addressed through discussions with Chunyan Wei to achieve consensus.

## 3 Results

### 3.1 Study selection

**Figure 1:**
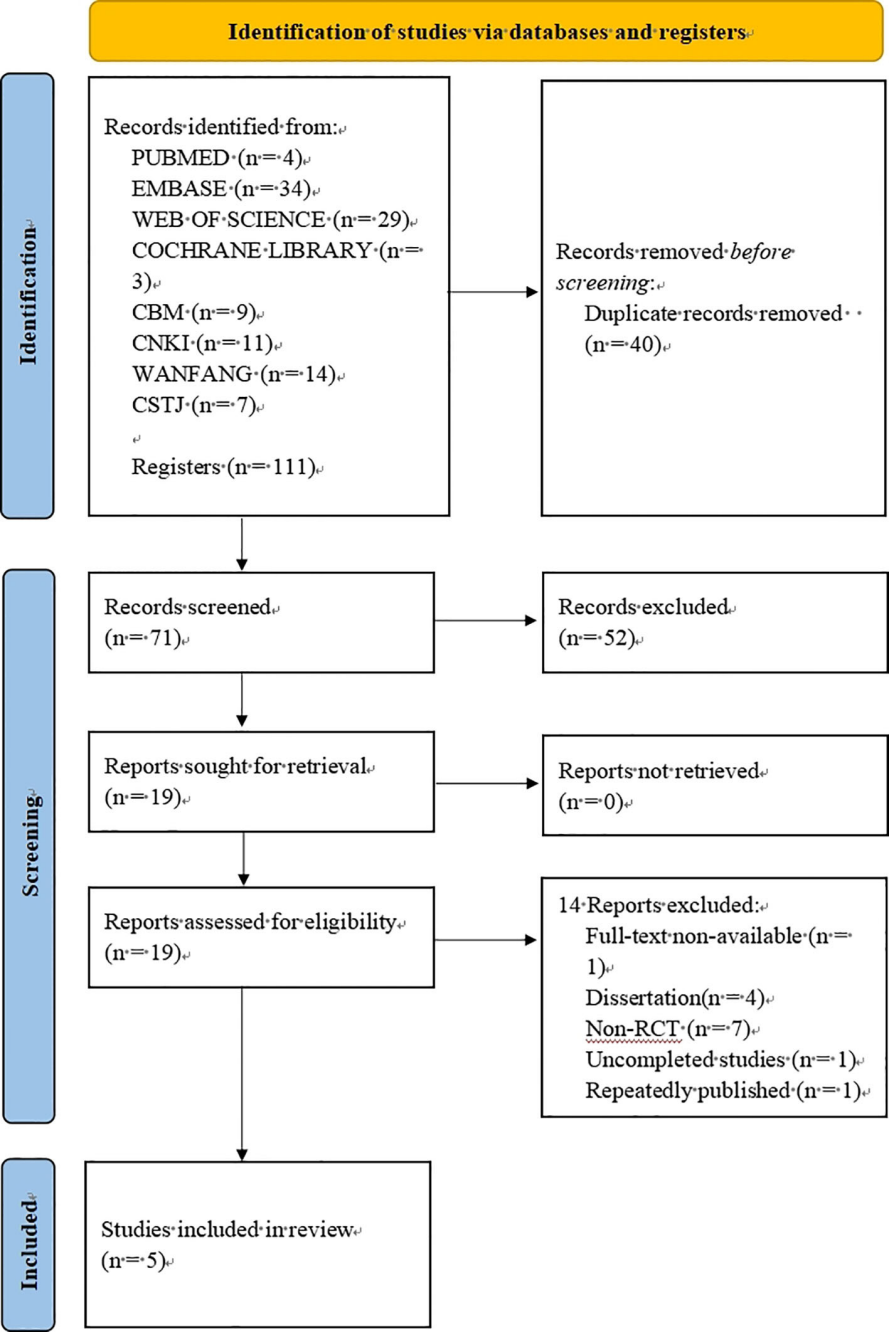
Study selection process.

Our systematic review identified 71 unique records after duplicate removal. Following an initial screening of titles and abstracts, 19 articles were deemed potentially eligible for full-text assessment. Applying our predefined exclusion criteria, we excluded 14 studies, resulting in 5 studies (11, 12, 27–29) that satisfied all inclusion criteria for the final meta-analysis (**Figure 1**).

### 3.2 Study characteristics

The primary features of the included studies are summarized in **Table 1**. Collectively, these five randomized controlled trials encompassed a total of 390 participants, with an average sample size of 78 ± 14.83.

**Table 1:** Characteristics of the included studies.

Study	Source	Study design	Test treatment			Control treatment			Country	Diagnostic criteria	Merger situations	Washout (days)	TMS parameters		Duration of treatment (weeks)	Concomitant antidepressant
			Participants(N)	Female (%)	Age (year)	Participants (N)	Female (%)	Age (year)					Stimulation regions	Frequency (Hz)		
XuY and HeQ 2024 (27)	Chinese Journal of Clinical Rational Drug Use	RCT	30	100	31.07± 7.70	30	100	30.58± 7.22	China	expert consensus	postpartum	first episode	Right Dorsolateral Prefrontal Cortex	1	6	SSRIs (Paroxetine)
SunM et al., 2023 (11)	Medical Innovation of China	RCT	40	50	36.96± 8.96	40	48	30.63± 6.63	China	ICD-10	treatment resistant	90	Left Temporal Parietal Cortex	1	8	SSRIs (Escitalopram)
HuangZ et al., 2024 (12)	Jiangxi Medical Journal	RCT	35	66	15.49± 1.20	35	29	15.63± 1.14	China	ICD-10	Adolescent	first episode	Left Dorsolateral Prefrontal Cortex	10	4	SSRIs (Fluoxetine)
JinN and TianL 2024	Journal of International Psychiatry	RCT	50	44	43.12± 6.44	50	40	42.68 ± 6.52	China	CCMD-3	insomnia	14	Left Dorsolateral Prefrontal Cortex	1	4	SNRIs (Venlafaxine)
WangJ, WenN, and ChenZ. 2024 (29)	Maternal and Child Health Care of China	RCT	40	100	26.85± 4.10	40	100	27.66 ± 4.21	China	expert consensus	postpartum	first episode	Dorsolateral Prefrontal Cortex	1	6	SNRIs (Venlafaxine)

TMS, transcranial magnetic stimulation; RCT, randomized clinical trial; ICD, International Classification of Diseases; CCMD, Chinese Classification of Mental Disorders; SSRIs, serotonin selective reuptake inhibitors; SNRIs, Serotonin-Norepinephrine Reuptake Inhibitors

### 3.3 Risk of bias

Utilizing the Cochrane ROB2 tool, we systematically assessed the risk of bias across the included studies (see **Supplementary Table S1**, **Supplementary Figure S1**), focusing on five critical domains. Concerning the randomization process, aside from the study by Wang et al. (29), which did not specify the randomization method in the original publication, all other studies explicitly reported using a randomized numeric table method for group allocation. Correspondence with the authors confirmed that Wang et al. also employed the random number table method, resulting in a classification of low risk for all studies regarding random sequence generation. Regarding interventions, although none of the studies implemented operator blinding, records indicated that interventions were conducted strictly according to established protocols without deviations, leading to a low risk assessment in this domain. All studies achieved comprehensive outcome data collection from randomized participants, with no attrition or missing data, and thus were rated as low risk for data completeness. In terms of outcome measurement, TSH assays across studies were performed by laboratory personnel blinded to group assignments, utilizing standardized laboratory procedures, which confer a low risk of measurement bias. Finally, for selective reporting, all studies adhered to predefined TSH testing protocols and reported complete data sets, with no evidence of selective outcome reporting, resulting in a low risk classification. Overall, the included studies demonstrated a low risk of bias across all key domains of the ROB2 assessment, supporting the robustness and reliability of the findings.

### 3.4 Meta-analysis of depression

**Figure 2:**
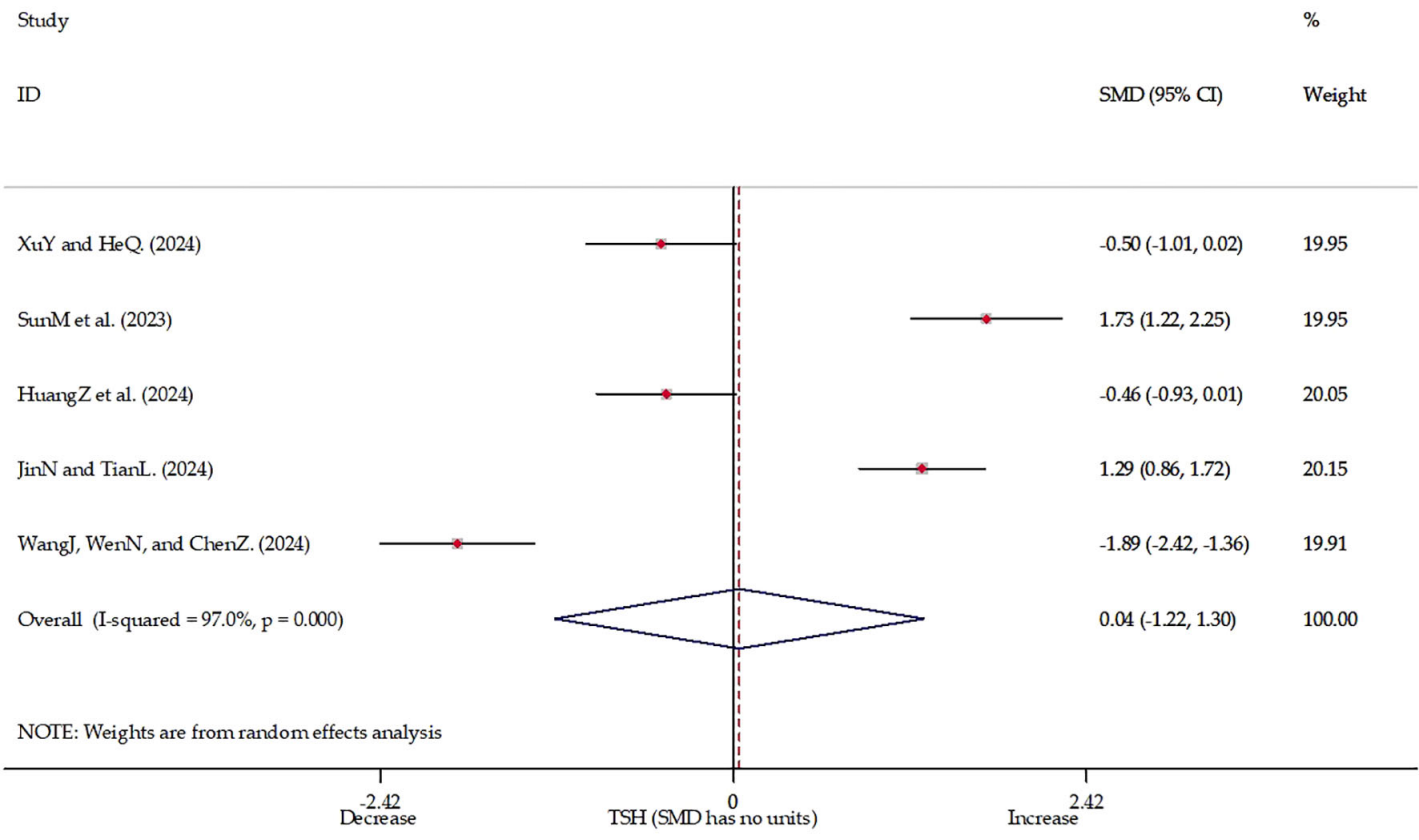
Forest plot of depression.

**Figure 3:**
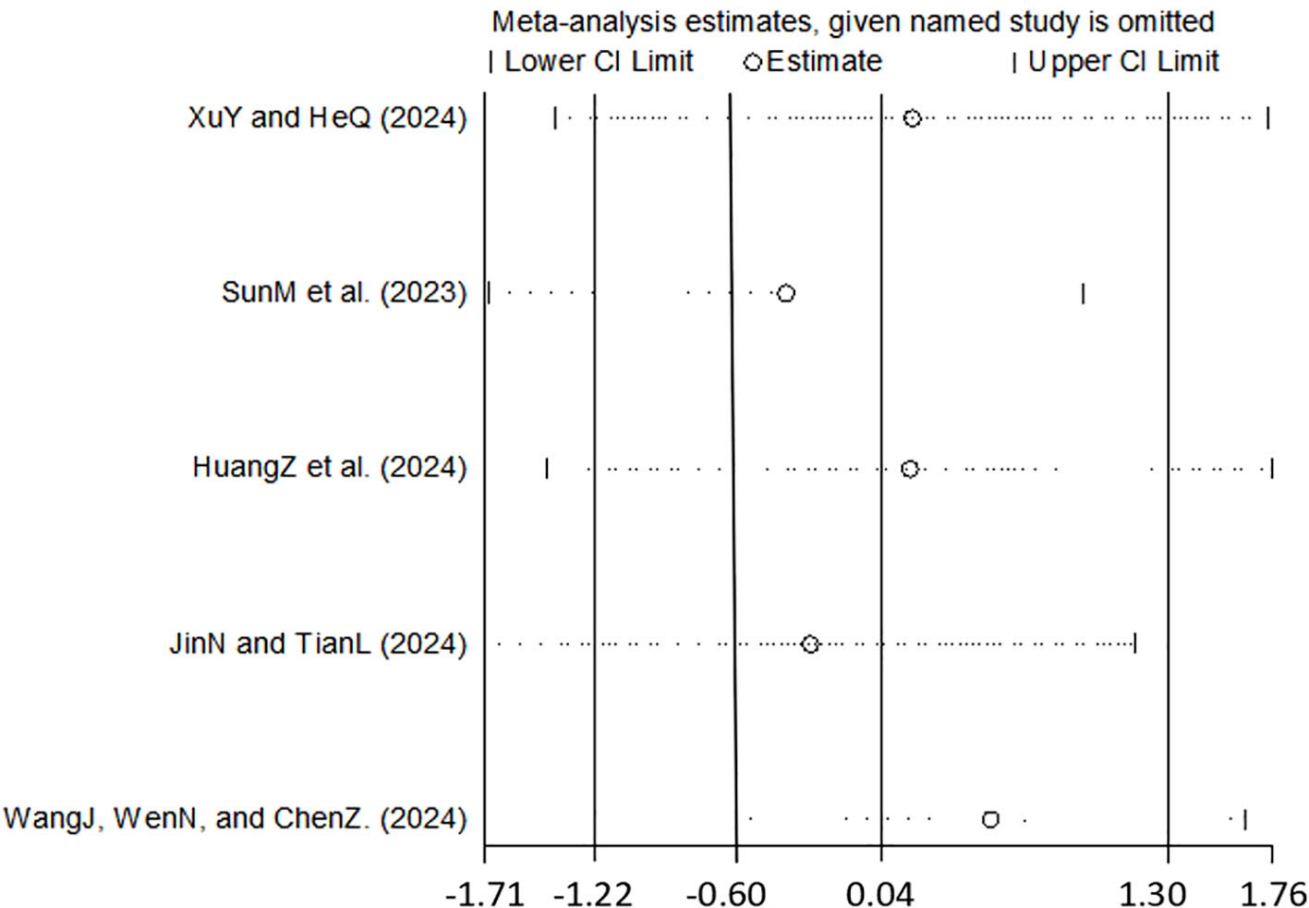
Leave-one-out method plot of depression.

Studies involving 390 participants reported TSH levels before and after TMS treatment in patients with depression. Significant heterogeneity was observed among the studies (I² = 97%, p < 0.001). Due to discrepancies in measurement units across studies, standardized mean difference effect size analyses were conducted. Since the SMD is a dimensionless parameter, the interpretation of SMD analysis results focuses on the directional trend of variation rather than the magnitude of the change (30–32). The meta-analysis (**Figure 2**, **Supplementary Figure S2**) indicated no statistically significant impact of TMS on TSH levels in depressed patients (Z = 0.06, p = 0.952). Visual inspection of funnel plots (**Supplementary Figure S3**) and Egger’s regression test (**Supplementary Figure S4, 5**, t = 1.01, p < 0.386) provided no evidence of publication bias. Meta-regression analysis (**Table 2**, **Supplementary Figure S6**) revealed that the number of females (p < 0.046) significantly contributed to heterogeneity, whereas age, sample size, treatment duration, TMS stimulation regions and frequency, and concomitant antidepressant medication did not produce significant effects. Sensitivity analyses employing a leave-one-out approach confirmed the robustness of the findings, as none of the four-study combinations reached statistical significance (all 95% confidence intervals included zero; **Figure 3**), thereby consistently supporting the primary conclusion.

**Table 2:** Meta-regression.

Covariates	Regression coefficients	Standard error	t	P>|t|	95% Confidence interval	
Number of women	-0.160	0.049	-3.290	0.046	-0.314	-0.005
Sample size	0.093	0.101	0.920	0.427	-0.229	0.415
Treatment duration (weeks)	0.166	0.499	0.330	0.761	-1.422	1.754
Age (years)	1.097	0.066	1.540	0.222	0.905	1.330
Stimulation regions	2.115	1.461	1.450	0.243	-2.534	6.764
Frequency (Hz)	-0.069	0.207	-0.330	0.760	-0.729	0.590
Concomitant antidepressant	-0.549	1.520	-0.360	0.742	-5.3844	4.287

### 3.5 Meta-analysis of postpartum depression

**Figure 4:**
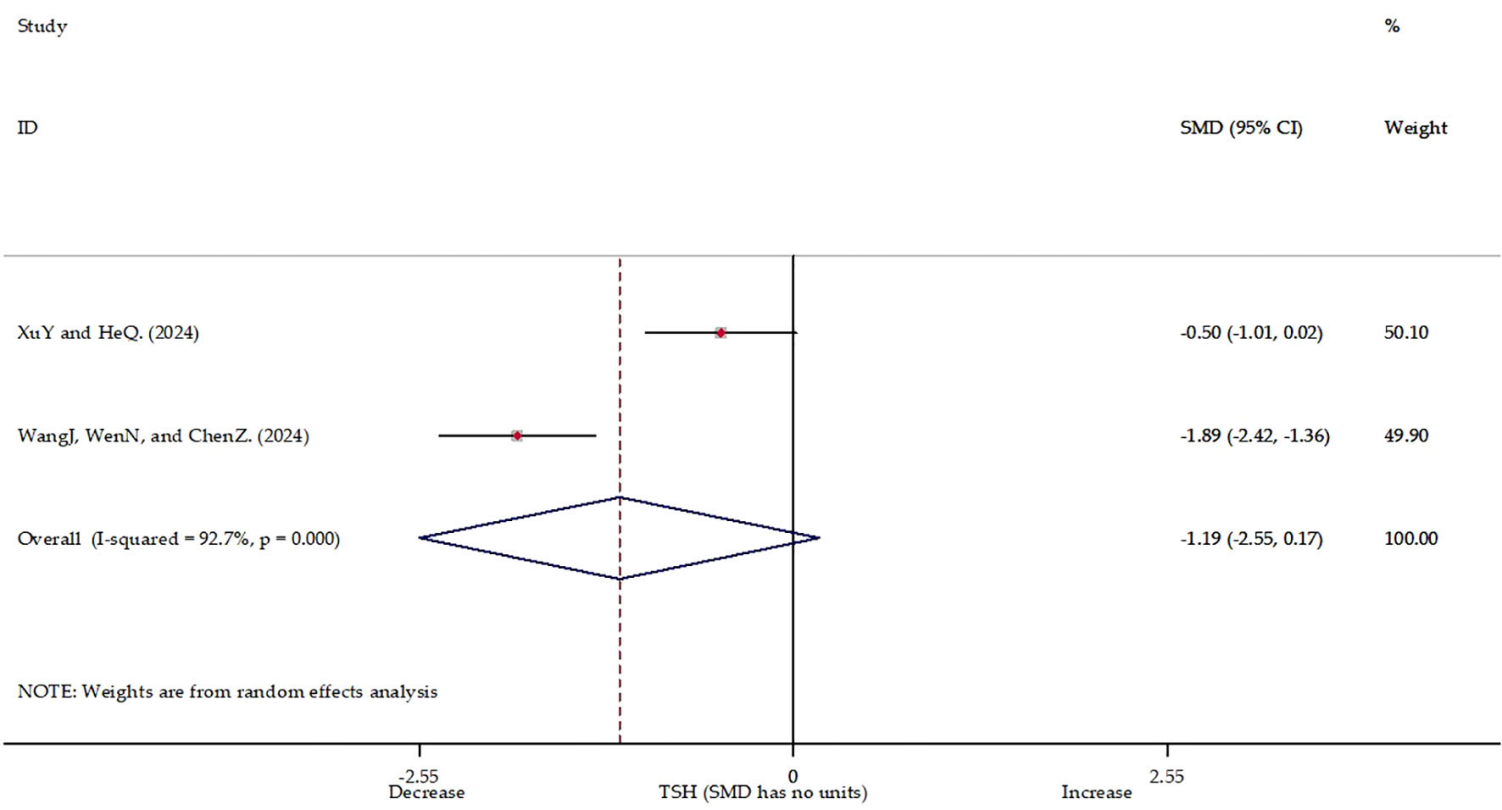
Forest plot of postpartum depression.

Based on the results of the meta-regression, we conducted a meta-analysis exclusively on studies that only include female participants. Our analysis encompassed two studies (27, 29) (n=140) assessing TSH levels in postpartum depression (PPD) patients before and after TMS therapy. It is important to note that this subgroup analysis is based on a very limited number of studies and participants. Significant heterogeneity was observed between studies (I²=92.7%, p<0.001), along with variability in measurement units. The forest plot analysis (**Figure 4**, **Supplementary Figure S7**) indicated no statistically significant effect of TMS on TSH levels (Z = 1.71, P = 0.087). Notably, the individual study results diverged, suggesting limited stability of the pooled estimate. Given the small number of studies, high heterogeneity, and the distinct physiological context of the postpartum period, which itself influences HPT axis dynamics (33, 34), these PPD-specific results should be interpreted with considerable caution. These findings underscore the necessity for further high-quality research to elucidate this potential association.

### 3.6 Grading of recommendations assessment

We assessed the Grading of Recommendations Assessment, Development, and Evaluation (GRADE) (35, 36) for the two meta-analyses, as detailed in **Table 3**. The depression group was rated as moderate quality, whereas the postpartum depression group was classified as low quality.

**Table 3:** GRADE evidence profile for included studies.

Group	Outcomes	Quality assessment							Number of patients		Effect	Quality
		Studies	Design	Risk of bias	Inconsistency	Indirectness	Imprecision	Other considerations	Test	Control	Absolute	
Depression	TSH	5	RCT	Not serious	Serious^a^	Not serious	Not serious	None	195	195	SMD 0.04 SD lower	⊕⊕⊕◯
											(1.30 lower to 1.22 higher)	Moderate
Postpartum Depression	TSH	2	RCT	Not serious	Serious^a^	Not serious	Serious^b^	None	70	70	SMD 1.19 SD lower	⊕⊕◯
											(2.55 lower to 0.17 higher)	Low

a: Downgrade by 1 level for I-squared (I²) exceeding 70%; b: The downgrade by 1 level is warranted due to the limited number of included studies, which leads to excessively broad confidence intervals; RCT: randomized controlled trial; SMD: standardized mean difference; SD: standard deviation.

## 4 Discussion

Our finding suggests that the antidepressant effects of TMS may not involve the HPT axis. The divergent results observed by Kito et al. (9) and Meille et al. (10) in treatment responders further indirectly indicate that changes in TSH levels are unrelated to the antidepressant efficacy of TMS. TMS exerts its antidepressant effects via multiple neurobiological pathways. Primarily, TMS exhibits anti-inflammatory properties and modulates microglial activity in depressive and anxiety disorders (37). It also influences 5-hydroxytryptamine (5-HT) receptor sensitivity (38). Moreover, TMS facilitates dopaminergic neurotransmission (7, 39) and promotes neuroplasticity by reorganizing neural circuits and functional brain networks (40, 41). Additionally, it enhances hippocampal neurogenesis and synaptic plasticity (42, 43). However, evidence regarding the neuromodulatory effects of TMS on the HPT axis remains limited. While some studies, such as Kito et al. (9), have proposed that TMS may indirectly influence the HPT axis by modulating limbic regions rich in TRH receptors (e.g., hippocampus and amygdala), thereby potentially affecting hypothalamic TRH and pituitary TSH release. Nevertheless, the overall evidence remains inconclusive. Crucially, the findings of the present meta-analysis, which aggregate results across multiple RCTs, do not support a statistically significant modulatory effect of TMS on TSH levels at the group level.

Notably, the lack of a significant modulatory effect of TMS on TSH levels observed in this meta-analysis may be explained by several neurophysiological considerations. Firstly, from a circuit anatomy perspective, the primary cortical targets of conventional TMS protocols for depression (e.g., the dorsolateral prefrontal cortex) have indirect and polysynaptic connections to the hypothalamus, the key regulator of the HPT axis. Consequently, neuromodulatory signals from TMS may be substantially attenuated before reaching the core of the HPT axis, in contrast to its more direct effects on cortical-limbic circuits (44, 45). Secondly, concerning the hierarchy of neuroendocrine systems, the HPT axis functions as a tonic, homeostatic system designed for long-term metabolic stability. This contrasts with phasic, stress-responsive systems like the hypothalamic-pituitary-adrenal (HPA) axis, which is more directly linked to depression-related circuits and may be more susceptible to acute interventions like TMS (46). Finally, the HPT axis itself is governed by a robust negative feedback loop. Emerging mathematical models suggest that this axis maintains FT3 homeostasis through intricate feedforward and feedback cascades, rendering it highly resistant to external perturbations; such inherent robustness and global stability may buffer against the subtle neuromodulatory signals potentially generated by TMS (47, 48).

The observed variations in TSH levels in some non-randomized controlled studies may be attributable to confounding factors. Research conducted by Abić Leko et al. (49) revealed that tobacco smoking is associated with a reduction in serum TSH levels, concomitant with elevations in circulating T3 and T4 concentrations. Additionally, body mass index (BMI) exhibited a positive correlation with both TSH and free T3 levels (50, 51). Excessive iodine intake was linked to increased TSH secretion and suppressed thyroid hormone synthesis (52, 53), whereas perchlorate exposure was correlated with impaired thyroid hormone biosynthesis (54). The meta-regression results suggest that the number of females may be a source of heterogeneity, potentially due to the greater influence of various factors on thyroid function in women compared to men. Pregnancy elevates maternal thyroid hormone requirements due to fetal development, increased placental deiodinase type 3 activity leading to enhanced thyroid hormone catabolism, augmented renal iodine excretion, and elevated thyroxine-binding globulin (TBG) concentrations. Human chorionic gonadotropin (HCG) exerts a thyrotropic effect, resulting in a reduced TSH reference range in pregnant women relative to non-pregnant counterparts. This TSH suppression may persist postpartum and has been linked to decreased TSH levels in women experiencing postpartum depression (33, 34). Furthermore, Ballinger et al. (55) reported that menstruating women aged 45 or older with depression exhibited significantly increased serum TSH and T3 levels compared to other groups. However, this study employs a randomized controlled trial meta-analysis, which minimizes the impact of confounding factors and yields higher-quality evidence.

The high statistical heterogeneity observed (I² = 97%, p < 0.001) is a key finding of this meta-analysis. While our meta-regression identified the number of female participants as a significant moderator, the substantial unexplained heterogeneity invites a thematic consideration of other plausible sources, which were likely underpowered in our quantitative analysis due to the limited number of studies. These can be grouped into three categories (1): Patient Population Characteristics, such as gender distribution and postpartum status where the inclusion of studies on postpartum depression introduces a distinct physiological context with altered HPT axis regulation (2); TMS Intervention Parameters, including variability in stimulation site (e.g., left vs. right DLPFC) and protocol details; and (3) Clinical Context, such as differences in concomitant antidepressant use. The confluence of these demographic, technical, and contextual factors likely underlies the observed heterogeneity, indicating that our pooled estimate averages across diverse clinical scenarios and underscoring the need for future RCTs with standardized designs to isolate specific effects.

To our knowledge, this constitutes the first systematic review and meta-analysis of existing randomized controlled trials investigating the association between TMS and TSH levels. However, several methodological limitations must be carefully considered when interpreting these results. Firstly, the combined sample size across the five included studies was relatively modest (n=390), with only 140 participants in the postpartum depression group, potentially limiting statistical power. Moreover, although we conducted a meta-regression analysis, we did not identify the source of high heterogeneity other than the number of females. This may be due to the low statistical power of moderator analysis in only five studies. In light of these limitations, our findings must be framed as preliminary. They should not be construed as conclusive evidence against an association, but rather as a result likely influenced by high heterogeneity and low statistical power. This underscores the imperative for future, well-powered RCTs with standardized protocols and pre-specified subgroup analyses to resolve this question. Secondly, due to the original research employing heterogeneous measurement units, we predominantly adopted the SMD as the aggregate effect size metric. The SMD normalizes effect sizes across studies by translating them into a unified scale, thereby reducing bias introduced by disparate measurement units. Nonetheless, as the SMD is a unitless measure, interpretation primarily emphasizes the directionality of effects rather than their magnitude. Furthermore, this study focused solely on TSH and did not encompass a comprehensive multidimensional assessment of the HPT axis. Future research and subsequent reviews should aim to incorporate a broader range of HPT axis hormones to build upon our findings and achieve a more holistic understanding. Importantly, despite comprehensive searches across key databases, all studies meeting the inclusion criteria were conducted within Chinese populations. Given that racial, dietary (iodine intake), and genetic differences may influence thyroid function and TMS responsiveness, the external validity of these findings is limited. Caution is warranted when extrapolating these results to other populations. Further randomized controlled trials are necessary to validate these findings in different geographic regions, such as Europe or Africa, and to explore potential racial or regional variations in the neuroendocrine effects of TMS.

In summary, this first systematic review and meta-analysis of RCTs synthesizes evidence that repetitive TMS does not exert a statistically significant modulatory effect on TSH levels at the group level in patients with depression. This core finding may be explained by the indirect neuroanatomical connectivity to the hypothalamus, the tonic homeostatic nature of the HPT axis, and its robust intrinsic feedback regulation. This supports the notion that the primary antidepressant mechanisms of TMS may operate independently of direct HPT axis modulation. However, these results should be interpreted as preliminary evidence of no significant effect, not as conclusive proof of no association. This is due to the inherent limitations of the current evidence base, specifically the small number of studies, limited sample size, and high heterogeneity. Therefore, to definitively elucidate the neuroendocrine dimensions of TMS therapy, future research must address the current limitations through concrete steps. We recommend the design of large, multicenter RCTs to achieve sufficient statistical power and demographic diversity. Such studies should implement and report standardized TMS protocols (e.g., specifying stimulation site, frequency, and pulse number) to minimize technical heterogeneity. Crucially, they should incorporate a comprehensive HPT-axis biomarker panel, including FT3 and FT4—and where feasible, TRH—to allow for a multidimensional assessment. Finally, given the influence of sex and reproductive status highlighted in our analysis, these trials should be adequately powered to conduct pre-specified, stratified analyses (e.g., by sex and postpartum status) to disentangle their specific effects.

## Data Availability

The original contributions presented in the study are included in the article/**Supplementary Material**. Further inquiries can be directed to the corresponding author.
