# Supplementary material for: The impact of transcranial magnetic stimulation on serum thyroid-stimulating hormone levels in depressive patients: a systematic review and meta-analysis of randomized controlled trials
Source: Front Psychiatry. 2026 Jan 22;17:1716377. doi: 10.3389/fpsyt.2026.1716377 (PMC12872799; doi:10.3389/fpsyt.2026.1716377)
Supplement: Supplementary file 1 [file DataSheet1.docx]

Supplementary Material

# Search strategy

## Database: Pubmed

| Date of search: November 27, 2025; Search results:4 | |  |
| --- | --- | --- |
| # | Searches | Results |
| #1 | "Depression"[Mesh] OR "Depressive Disorder"[Mesh] OR "Depression, Postpartum"[Mesh] OR "Depressive Disorder, Treatment-Resistant"[Mesh] OR "Depressive Disorder, Major"[Mesh] OR "Major Depressive Disorder 1" [Supplementary Concept] OR "Major Depressive Disorder 2" [Supplementary Concept] | 288,275 |
| #2 | (((((((((((((((((((((((((((((((((((((((Depressive Symptoms[Title/Abstract]) OR (Depressive Symptom[Title/Abstract])) OR (Emotional Depression[Title/Abstract])) OR (Depressive Disorders[Title/Abstract])) OR (Depressive Neuroses[Title/Abstract])) OR (Depressive Neurosis[Title/Abstract])) OR (Endogenous Depression[Title/Abstract])) OR (Endogenous Depressions[Title/Abstract])) OR (Depressive Syndrome[Title/Abstract])) OR (Depressive Syndromes[Title/Abstract])) OR (Syndrome, Depressive[Title/Abstract])) OR (Neurotic Depression[Title/Abstract])) OR (Neurotic Depressions[Title/Abstract])) OR (Melancholia[Title/Abstract])) OR (Melancholias[Title/Abstract])) OR (Unipolar Depression[Title/Abstract])) OR (Unipolar Depressions[Title/Abstract])) OR (Postnatal Depression[Title/Abstract])) OR (Post-Partum Depression[Title/Abstract])) OR (Post Partum Depression[Title/Abstract])) OR (Postpartum Depression[Title/Abstract])) OR (Post-Natal Depression[Title/Abstract])) OR (Post Natal Depression[Title/Abstract])) OR (Postnatal Dysphoria[Title/Abstract])) OR (Post-Partum Dysphoria[Title/Abstract])) OR (Post Partum Dysphoria[Title/Abstract])) OR (Postpartum Dysphoria[Title/Abstract])) OR (Post-Natal Dysphoria[Title/Abstract])) OR (Post Natal Dysphoria[Title/Abstract])) OR (Treatment-Resistant Depressive Disorder[Title/Abstract])) OR (Treatment-Resistant Depressive Disorders[Title/Abstract])) OR (Refractory Depression[Title/Abstract])) OR (Refractory Depressions[Title/Abstract])) OR (Therapy-Resistant Depression[Title/Abstract])) OR (Therapy Resistant Depression[Title/Abstract])) OR (Therapy-Resistant Depressions[Title/Abstract])) OR (Treatment Resistant Depression[Title/Abstract])) OR (Treatment Resistant Depressions[Title/Abstract])) OR (Major Depressive Disorders[Title/Abstract])) OR (Major Depressive Disorder[Title/Abstract]) | 146,137 |
| #3 | "Thyrotropin"[Mesh] OR "TSHR protein, human" [Supplementary Concept] | 35,153 |
| #4 | ((((Thyroid Stimulating Hormone[Title/Abstract]) OR (Thyrotrophin[Title/Abstract])) OR (Thyroid Stimulating Hormone[Title/Abstract])) OR (Thyreotropin[Title/Abstract])) OR (TSH[Title/Abstract]) | 46,101 |
| #5 | "Transcranial Magnetic Stimulation"[Mesh] | 17,522 |
| #6 | (((((((Magnetic Stimulations, Transcranial[Title/Abstract]) OR (Magnetic Stimulation, Transcranial[Title/Abstract])) OR (Stimulations, Transcranial Magnetic[Title/Abstract])) OR (Stimulation, Transcranial Magnetic[Title/Abstract])) OR (Transcranial Magnetic Stimulations[Title/Abstract])) OR (Transcranial Magnetic Stimulation, Paired Pulse[Title/Abstract])) OR (Transcranial Magnetic Stimulation, Repetitive[Title/Abstract])) OR (Transcranial Magnetic Stimulation, Single Pulse[Title/Abstract]) | 24,959 |
| #7 | #1 OR #2 | 339,120 |
| #8 | #3 OR #4 | 59,160 |
| #9 | #5 OR #6 | 27,161 |
| #10 | #7 AND #8 AND #9 | 4 |

## Database: Embase

| Date of search: November 27, 2025; Search results: 34 | |  | |  |
| --- | --- | --- | --- | --- |
| # | Searches | | Results | |
| #1 | 'depression'/exp | | 1,104,871 | |
| #2 | 'central depression':ab,ti OR 'clinical depression':ab,ti OR 'depressive disease':ab,ti OR 'depressive disorder':ab,ti OR 'depressive episode':ab,ti OR 'depressive illness':ab,ti OR 'depressive personality disorder':ab,ti OR 'depressive state':ab,ti OR 'depressive symptom':ab,ti OR 'depressive syndrome':ab,ti OR 'mental depression':ab,ti OR 'parental depression':ab,ti OR 'depression':ab,ti | | 724,507 | |
| #3 | thyrotropin:ab,ti | | 21,566 | |
| #4 | 'actyron':ab,ti OR 'dermathycin':ab,ti OR 'pretiron':ab,ti OR 'thyreoid stimulating hormone':ab,ti OR 'thyroid stimulating hormone':ab,ti OR 'thyroid stimulation hormone':ab,ti OR 'thyropar':ab,ti OR 'thyrostimulating hormone':ab,ti OR 'thyrostimulin':ab,ti OR 'thyrotropar':ab,ti OR 'thyrotrophic hormone':ab,ti OR 'thyrotrophin':ab,ti OR 'thyrotropic hormone':ab,ti OR 'thyrotropine':ab,ti OR 'thyrtropar':ab,ti OR 'thytropar':ab,ti OR 'tsh':ab,ti OR 'thyrotropin' | | 124,334 | |
| #5 | 'transcranial magnetic stimulation'/exp | | 41,737 | |
| #6 | 'Magnetic Stimulations, Transcranial':ab,ti OR 'Magnetic Stimulation, Transcranial':ab,ti OR 'Stimulations, Transcranial Magnetic':ab,ti OR 'Stimulation, Transcranial Magnetic':ab,ti OR 'Transcranial Magnetic Stimulations':ab,ti OR 'Transcranial Magnetic Stimulation, Paired Pulse':ab,ti OR 'Transcranial Magnetic Stimulation, Repetitive':ab,ti OR 'Transcranial Magnetic Stimulation, Single Pulse' | | 395 | |
| #7 | #1 OR #2 | | 1,107,610 | |
| #8 | #3 OR #4 | | 124,334 | |
| #9 | #5 OR #6 | | 41,771 | |
| #10 | #7 AND #8 AND #9 | | 34 | |

## Database: Web of science

| Date of search: November 27, 2025; Search results:29 | | |
| --- | --- | --- |
| # | Searches | Results |
| #1 | TS=(depression OR central depression OR clinical depression OR depressive disease OR depressive disorder OR depressive episode OR depressive illness OR depressive personality disorder OR depressive state OR depressive symptom OR depressive syndrome OR mental depression OR parental depression OR depression) | 1,053,955 |
| #2 | TS=(thyrotropin OR actyron OR dermathycin OR pretiron OR thyreoid stimulating hormone OR thyroid stimulating hormone OR thyroid stimulation hormone OR thyropar OR thyrostimulating hormone OR thyrostimulin OR thyrotropar OR thyrotrophic hormone OR thyrotrophin OR thyrotropic hormone OR thyrotropine OR thyrtropar OR thytropar OR tsh OR thyrotropin) | 65,857 |
| #3 | TS=(transcranial magnetic stimulation OR Magnetic Stimulations, Transcranial OR Magnetic Stimulation, Transcranial OR Stimulations, Transcranial Magnetic OR Stimulation, Transcranial Magnetic OR Transcranial Magnetic Stimulations OR Transcranial Magnetic Stimulation, Paired Pulse OR Transcranial Magnetic Stimulation, Repetitive OR Transcranial Magnetic Stimulation, Single Pulse) | 43,334 |
| #4 | #1 AND #2 AND #3 | 29 |

## Database: Cochrane Library

| Date of search: November 27, 2025; Search results: 3 | | |
| --- | --- | --- |
| # | Searches | Results |
| #1 | MeSH descriptor: [Depression] explode all trees OR MeSH descriptor: [Depressive Disorder] explode all trees OR MeSH descriptor: [Depression, Postpartum] explode all trees OR MeSH descriptor: [Depressive Disorder, Treatment-Resistant] explode all trees OR MeSH descriptor: [Depressive Disorder, Major] explode all trees OR (Depressive Symptoms):ti,ab,kw OR (Depressive Symptom):ti,ab,kw OR (Emotional Depression):ti,ab,kw OR (Depressive Disorders):ti,ab,kw OR (Depressive Neuroses):ti,ab,kw OR (Depressive Neurosis):ti,ab,kw OR (Endogenous Depression):ti,ab,kw OR (Endogenous Depressions):ti,ab,kw OR (Depressive Syndrome):ti,ab,kw OR (Depressive Syndromes):ti,ab,kw OR (Syndrome, Depressive):ti,ab,kw OR (Neurotic Depression):ti,ab,kw OR (Neurotic Depressions):ti,ab,kw OR (Melancholia):ti,ab,kw OR (Melancholias):ti,ab,kw OR (Unipolar Depression):ti,ab,kw OR (Unipolar Depressions):ti,ab,kw OR (Postnatal Depression):ti,ab,kw OR (Post-Partum Depression):ti,ab,kw OR (Post Partum Depression):ti,ab,kw OR (Postpartum Depression):ti,ab,kw OR (Post-Natal Depression):ti,ab,kw OR (Post Natal Depression):ti,ab,kw OR (Postnatal Dysphoria):ti,ab,kw OR (Post-Partum Dysphoria):ti,ab,kw OR (Post Partum Dysphoria):ti,ab,kw OR (Postpartum Dysphoria):ti,ab,kw OR (Post-Natal Dysphoria):ti,ab,kw OR (Post Natal Dysphoria):ti,ab,kw OR (Treatment-Resistant Depressive Disorder):ti,ab,kw OR (Treatment-Resistant Depressive Disorders):ti,ab,kw OR (Refractory Depression):ti,ab,kw OR (Refractory Depressions):ti,ab,kw OR (Therapy-Resistant Depression):ti,ab,kw OR (Therapy Resistant Depression):ti,ab,kw OR (Therapy-Resistant Depressions):ti,ab,kw OR (Treatment Resistant Depression):ti,ab,kw OR (Treatment Resistant Depressions):ti,ab,kw OR (Major Depressive Disorders):ti,ab,kw OR (Major Depressive Disorder):ti,ab,kw | 60,596 |
| #2 | MeSH descriptor: [Thyrotropin] explode all trees OR (Thyroid Stimulating Hormone):ti,ab,kw OR (Thyrotrophin):ti,ab,kw OR (Thyroid Stimulating Hormone):ti,ab,kw OR (Thyreotropin):ti,ab,kw OR (TSH):ti,ab,kw | 4,105 |
| #3 | MeSH descriptor: [Transcranial Magnetic Stimulation] explode all trees | 2930 |
| #4 | (Magnetic Stimulations, Transcranial):ti,ab,kw OR (Magnetic Stimulation, Transcranial):ti,ab,kw OR (Stimulations, Transcranial Magnetic):ti,ab,kw OR (Stimulation, Transcranial Magnetic):ti,ab,kw OR (Transcranial Magnetic Stimulations):ti,ab,kw OR (Transcranial Magnetic Stimulation, Paired Pulse):ti,ab,kw OR (Transcranial Magnetic Stimulation, Repetitive):ti,ab,kw OR (Transcranial Magnetic Stimulation, Single Pulse):ti,ab,kw | 9,761 |
| #5 | #3 OR #4 | 9,761 |
| #6 | #1 AND #2 AND #5 | 3 |

## Database: China Biology Medicine disc (CBM)

| Date of search: November 27, 2025; Search results: 9 | | |
| --- | --- | --- |
| # | Searches | Results |
| #1 | "抑郁症"[不加权:扩展] | 56,874 |
| #2 | "抑郁综合征"[常用字段:智能] OR "抑郁症,神经官能性"[常用字段:智能] OR "神经官能性抑郁症"[常用字段:智能] OR "忧郁症"[常用字段:智能] OR "单相抑郁症"[常用字段:智能] OR "抑郁性神经症"[常用字段:智能] OR "抑郁症,内因性"[常用字段:智能] OR "内源性抑郁症"[常用字段:智能] OR "内因性抑郁症"[常用字段:智能] | 73,733 |
| #3 | "促甲状腺素"[不加权:扩展] | 8,389 |
| #4 | "促甲状腺激素"[常用字段:智能] OR "TSH"[常用字段:智能] OR "促甲状腺素"[常用字段:智能] | 23,663 |
| #5 | "经颅磁刺激"[不加权:扩展] | 5,836 |
| #6 | "经颅磁刺激,单脉冲"[常用字段:智能] OR "经颅磁刺激,成对脉冲"[常用字段:智能] OR "经颅磁刺激,重复"[常用字段:智能] | 5 |
| #7 | (#1) OR (#2) | 73,733 |
| #8 | (#3) OR (#4) | 23,663 |
| #9 | (#5) OR (#6) | 5,837 |
| #10 | (#7) AND (#8) AND (#9) | 9 |

## Database: China National Knowledge Infrastructure (CNKI)

| Date of search: November 27, 2025; Search results: 11 | | |
| --- | --- | --- |
|  | Searches | Results |
|  | (TKA = 抑郁症 OR TKA = 抑郁综合征 OR TKA = 抑郁症,神经官能性 OR TKA = 神经官能性抑郁症 OR TKA = 忧郁症 OR TKA = 单相抑郁症 OR TKA = 抑郁性神经症 OR TKA = 抑郁症,内因性 OR TKA = 内源性抑郁症 OR TKA = 内因性抑郁症) AND (TKA = 促甲状腺素 OR TKA = 促甲状腺激素 OR TKA = TSH OR TKA = 促甲状腺素) AND (TKA = 经颅磁刺激 OR TKA = 经颅磁刺激,单脉冲 OR TKA = 经颅磁刺激,成对脉冲 OR TKA = 经颅磁刺激,重复) | 11 |

## Database: Wanfang Data

| Date of search: November 27, 2025; Search results: 14 | | |
| --- | --- | --- |
|  | Searches | Results |
|  | 全部:(抑郁症 OR 抑郁综合征 OR 抑郁症,神经官能性 OR 神经官能性抑郁症 OR 忧郁症 OR 单相抑郁症 OR 抑郁性神经症 OR 抑郁症,内因性 OR 内源性抑郁症 OR 内因性抑郁症) and 全部:(促甲状腺素 OR 促甲状腺激素 OR TSH OR 促甲状腺素) and 全部:(经颅磁刺激 OR 经颅磁刺激,单脉冲 OR 经颅磁刺激,成对脉冲 OR 经颅磁刺激,重复) | 14 |

## Database: China Science and Technology Journal Database (CSTJ)

| Date of search: November 27, 2025; Search results: 7 | | |
| --- | --- | --- |
|  | Searches | Results |
|  | ([((((((((((任意字段=抑郁症 OR 任意字段=抑郁综合征) OR 任意字段=抑郁症,神经官能性) OR 任意字段=神经官能性抑郁症) OR 任意字段=忧郁症) OR 任意字段=单相抑郁症) OR 任意字段=抑郁性神经症) OR 任意字段=抑郁症,内因性) OR 任意字段=内源性抑郁症) OR 任意字段=内因性抑郁症) AND (((任意字段=促甲状腺素 OR 任意字段=促甲状腺激素) OR 任意字段=TSH) OR 任意字段=促甲状腺素)) AND (((任意字段=经颅磁刺激 OR 任意字段=经颅磁刺激,单脉冲) OR 任意字段=经颅磁刺激,成对脉冲) OR 任意字段=经颅磁刺激,重复))](https://qikan.cqvip.com/Qikan/search/index?LngMySearHistoryIdGuid=25495d3f-dd61-4a18-a5ba-15375c329f7a&from=Qikan_Article_History) | 7 |

# Supplementary Figures and Tables

## Supplementary Figures


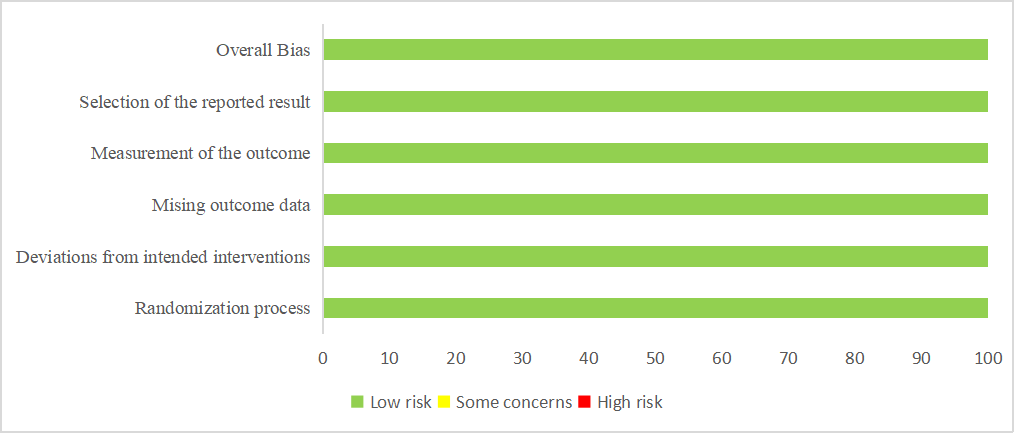


**Supplementary Figure 1.** Risk of bias (graphic display of risk of bias assessment).


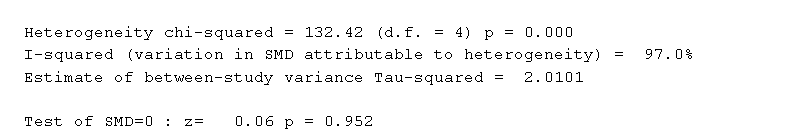


**Supplementary Figure 2.** Information on the forest plot of depression.


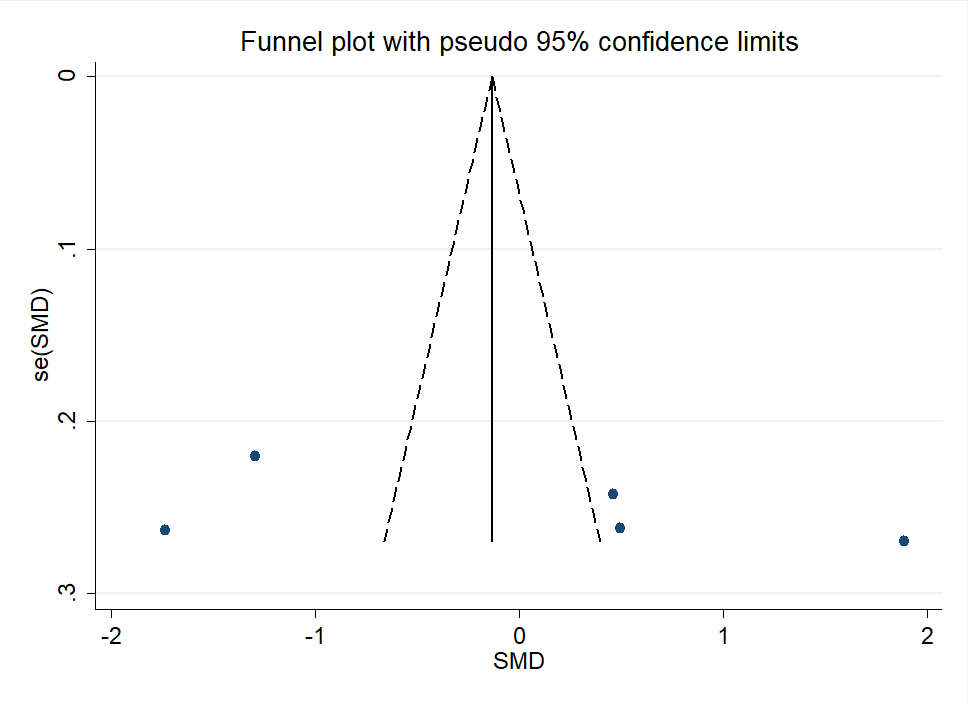


**Supplementary Figure 3.** Funnel plot of depression.


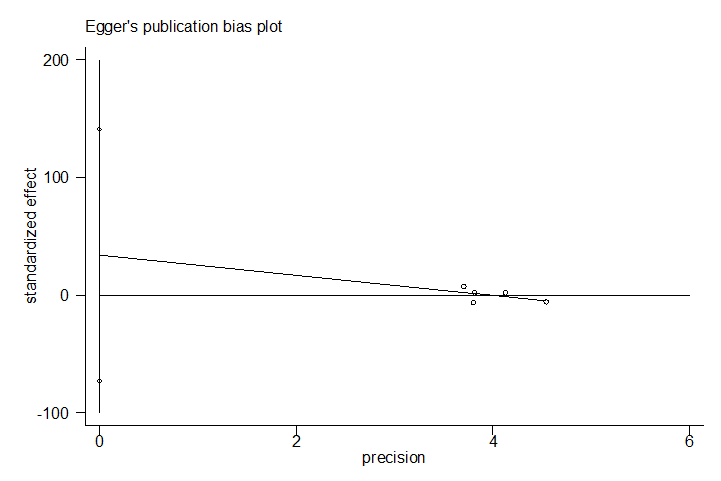


**Supplementary Figure 4 .** Egger plot of depression.


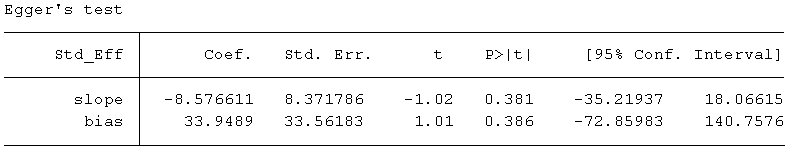


**Supplementary Figure 5.** Egger test of depression.


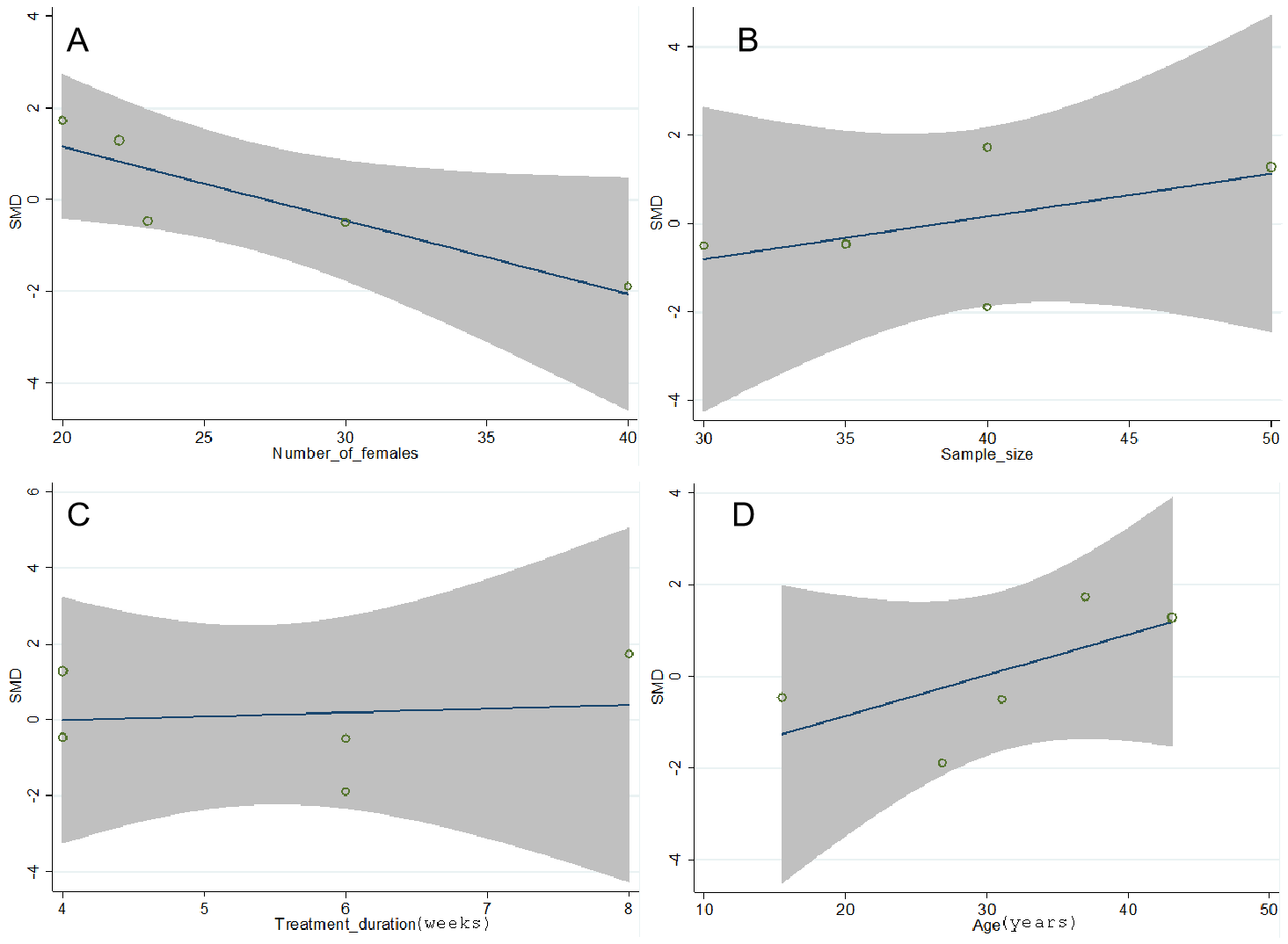


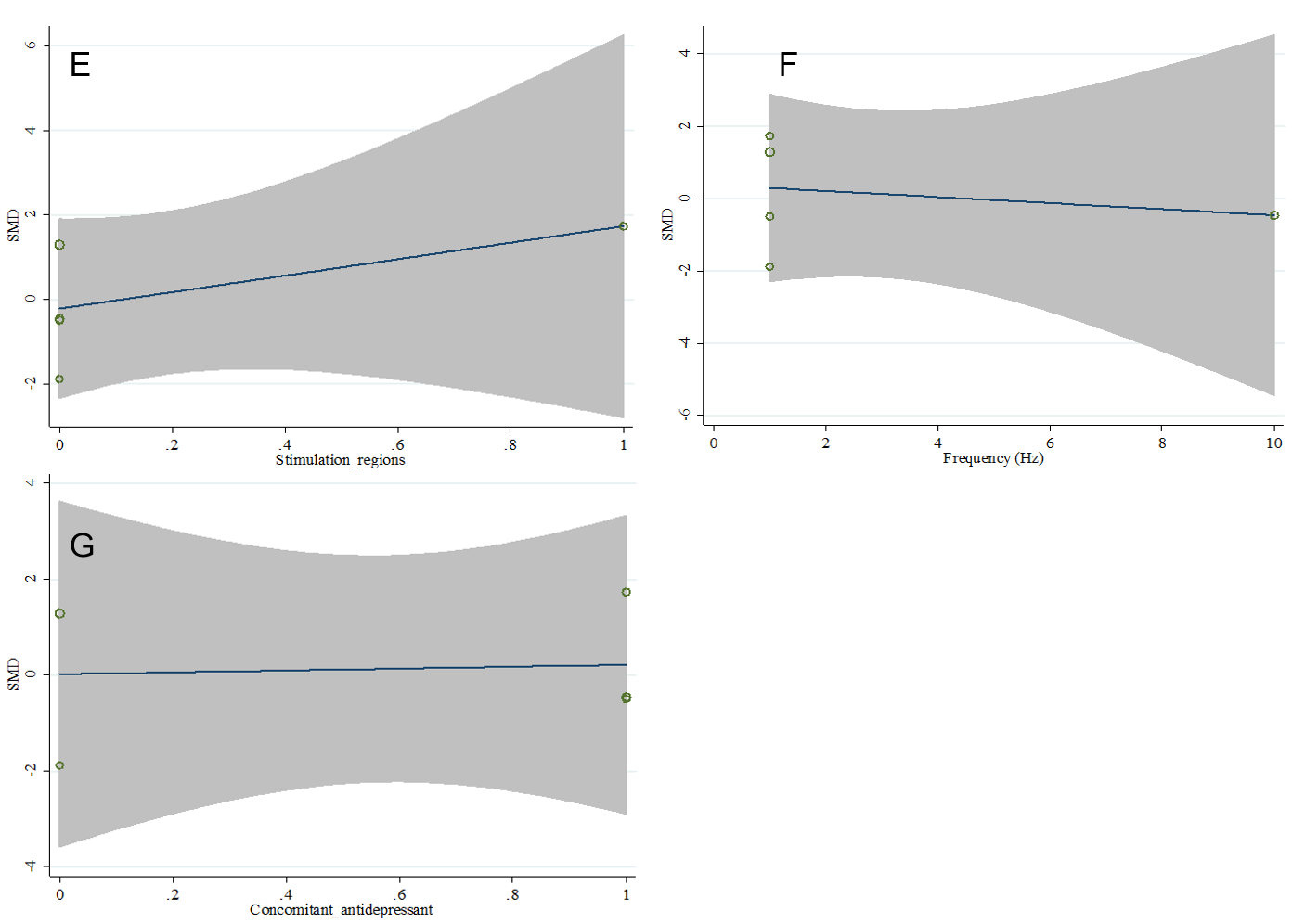


**Supplementary Figure 6.** Meta-regression plots of depression.

**
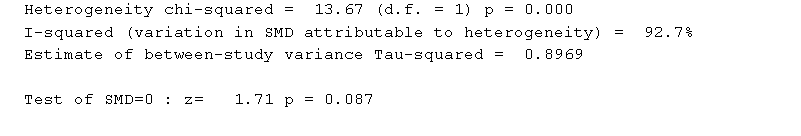
**

**Supplementary Figure 7.** Information on the forest plot of postpartum depression.

## Supplementary Tables

**Supplementary Table 1.** Risk of bias.


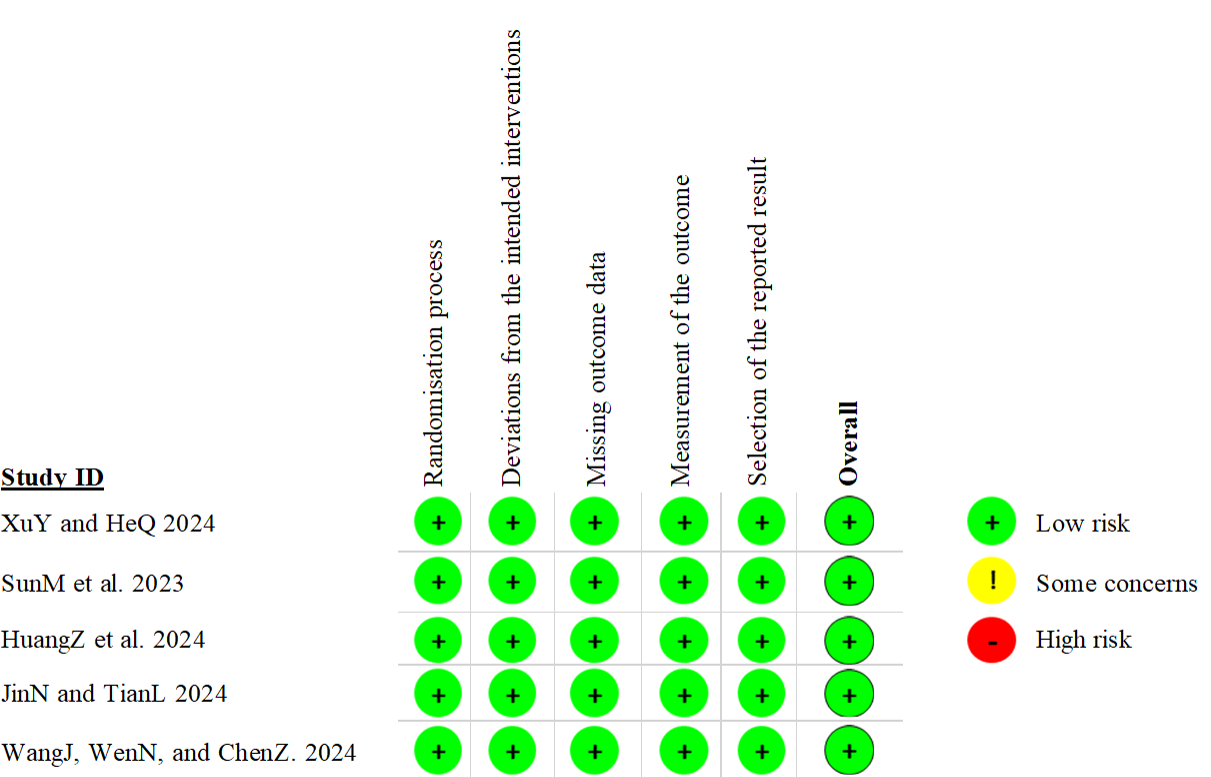


# **PRISMA 2020 checklist**

| **Section and Topic** | **Item #** | **Checklist item** | **Location where item is reported** |
| --- | --- | --- | --- |
| **TITLE** | | |  |
| Title | 1 | Identify the report as a systematic review. | **Title page** |
| **ABSTRACT** | | |  |
| Abstract | 2 | See the PRISMA 2020 for Abstracts checklist. | **lines 10 - 33** |
| **INTRODUCTION** | | |  |
| Rationale | 3 | Describe the rationale for the review in the context of existing knowledge. | **lines 35-65** |
| Objectives | 4 | Provide an explicit statement of the objective(s) or question(s) the review addresses. | **lines 66-70** |
| **METHODS** | | |  |
| Eligibility criteria | 5 | Specify the inclusion and exclusion criteria for the review and how studies were grouped for the syntheses. | **lines 72-79** |
| Information sources | 6 | Specify all databases, registers, websites, organisations, reference lists and other sources searched or consulted to identify studies. Specify the date when each source was last searched or consulted. | **Lines 80-92** |
| Search strategy | 7 | Present the full search strategies for all databases, registers and websites, including any filters and limits used. | **Supplementary_Material** |
| Selection process | 8 | Specify the methods used to decide whether a study met the inclusion criteria of the review, including how many reviewers screened each record and each report retrieved, whether they worked independently, and if applicable, details of automation tools used in the process. | **lines 80-92** |
| Data collection process | 9 | Specify the methods used to collect data from reports, including how many reviewers collected data from each report, whether they worked independently, any processes for obtaining or confirming data from study investigators, and if applicable, details of automation tools used in the process. | **lines 94-100** |
| Data items | 10a | List and define all outcomes for which data were sought. Specify whether all results that were compatible with each outcome domain in each study were sought (e.g. for all measures, time points, analyses), and if not, the methods used to decide which results to collect. | **lines 94-100** |
|  | 10b | List and define all other variables for which data were sought (e.g. participant and intervention characteristics, funding sources). Describe any assumptions made about any missing or unclear information. |  |
| Study risk of bias assessment | 11 | Specify the methods used to assess risk of bias in the included studies, including details of the tool(s) used, how many reviewers assessed each study and whether they worked independently, and if applicable, details of automation tools used in the process. | **lines 116-122** |
| Effect measures | 12 | Specify for each outcome the effect measure(s) (e.g. risk ratio, mean difference) used in the synthesis or presentation of results. | **lines 101-115** |
| Synthesis methods | 13a | Describe the processes used to decide which studies were eligible for each synthesis (e.g. tabulating the study intervention characteristics and comparing against the planned groups for each synthesis (item #5)). |  |
|  | 13b | Describe any methods required to prepare the data for presentation or synthesis, such as handling of missing summary statistics, or data conversions. |  |
|  | 13c | Describe any methods used to tabulate or visually display results of individual studies and syntheses. |  |
|  | 13d | Describe any methods used to synthesize results and provide a rationale for the choice(s). If meta-analysis was performed, describe the model(s), method(s) to identify the presence and extent of statistical heterogeneity, and software package(s) used. |  |
|  | 13e | Describe any methods used to explore possible causes of heterogeneity among study results (e.g. subgroup analysis, meta-regression). |  |
|  | 13f | Describe any sensitivity analyses conducted to assess robustness of the synthesized results. |  |
| Reporting bias assessment | 14 | Describe any methods used to assess risk of bias due to missing results in a synthesis (arising from reporting biases). | **lines 116-122** |
| Certainty assessment | 15 | Describe any methods used to assess certainty (or confidence) in the body of evidence for an outcome. | **Lines 101-115** |
| **RESULTS** | | |  |
| Study selection | 16a | Describe the results of the search and selection process, from the number of records identified in the search to the number of studies included in the review, ideally using a flow diagram. | **Lines 125-129** |
|  | 16b | Cite studies that might appear to meet the inclusion criteria, but which were excluded, and explain why they were excluded. |  |
| Study characteristics | 17 | Cite each included study and present its characteristics. | **Lines 130-133** |
| Risk of bias in studies | 18 | Present assessments of risk of bias for each included study. | **Lines 134-152** |
| Results of individual studies | 19 | For all outcomes, present, for each study: (a) summary statistics for each group (where appropriate) and (b) an effect estimate and its precision (e.g. confidence/credible interval), ideally using structured tables or plots. | **Lines 153-181** |
| Results of syntheses | 20a | For each synthesis, briefly summarise the characteristics and risk of bias among contributing studies. |  |
|  | 20b | Present results of all statistical syntheses conducted. If meta-analysis was done, present for each the summary estimate and its precision (e.g. confidence/credible interval) and measures of statistical heterogeneity. If comparing groups, describe the direction of the effect. |  |
|  | 20c | Present results of all investigations of possible causes of heterogeneity among study results. |  |
|  | 20d | Present results of all sensitivity analyses conducted to assess the robustness of the synthesized results. |  |
| Reporting biases | 21 | Present assessments of risk of bias due to missing results (arising from reporting biases) for each synthesis assessed. |  |
| Certainty of evidence | 22 | Present assessments of certainty (or confidence) in the body of evidence for each outcome assessed. | **Lines 182-185** |
| **DISCUSSION** | | |  |
| Discussion | 23a | Provide a general interpretation of the results in the context of other evidence. | **Lines 188-236** |
|  | 23b | Discuss any limitations of the evidence included in the review. | **Lines 237-274** |
|  | 23c | Discuss any limitations of the review processes used. |  |
|  | 23d | Discuss implications of the results for practice, policy, and future research. | **Lines 275-292** |
| **OTHER INFORMATION** | | |  |
| Registration and protocol | 24a | Provide registration information for the review, including register name and registration number, or state that the review was not registered. | **Lines 88-89** |
|  | 24b | Indicate where the review protocol can be accessed, or state that a protocol was not prepared. |  |
|  | 24c | Describe and explain any amendments to information provided at registration or in the protocol. |  |
| Support | 25 | Describe sources of financial or non-financial support for the review, and the role of the funders or sponsors in the review. | **Funding information** |
| Competing interests | 26 | Declare any competing interests of review authors. | **Lines 293-295** |
| Availability of data, code and other materials | 27 | Report which of the following are publicly available and where they can be found: template data collection forms; data extracted from included studies; data used for all analyses; analytic code; any other materials used in the review. | **Data availability and sources** |

*From:*  Page MJ, McKenzie JE, Bossuyt PM, Boutron I, Hoffmann TC, Mulrow CD, et al. The PRISMA 2020 statement: an updated guideline for reporting systematic reviews. BMJ 2021;372:n71. doi: 10.1136/bmj.n71

For more information, visit: <http://www.prisma-statement.org/>
